# Problematic Internet Use in Frontotemporal Dementia: A Case Series

**DOI:** 10.1002/acn3.70417

**Published:** 2026-05-19

**Authors:** Daniele Urso, Giuseppe Volpe, Agnese Valguarnera, Davide Vilella, Alessandra Vitulli, Valentina Gnoni, Alessia Giugno, Eleonora Rollo, Mark D. Griffiths, Giancarlo Logroscino

**Affiliations:** ^1^ Center for Neurodegenerative Diseases and the Aging Brain, Department of Clinical Research in Neurology University of Bari “Aldo Moro”, “Pia Fondazione Cardinale G. Panico” Tricase Italy; ^2^ Department of Translational Biomedicine and Neuroscience University of Bari “Aldo Moro” Bari Italy; ^3^ Department of Neurosciences Università Cattolica del Sacro Cuore Rome Italy; ^4^ International Gaming Research Unit, Psychology Department Nottingham Trent University Nottingham UK

**Keywords:** frontotemporal dementia, online addictions, problematic internet use

## Abstract

The present study investigated problematic internet use (PIU) among 61 patients with frontotemporal dementia (FTD) compared to a cohort of 354 patients with mild cognitive impairment (MCI) and Alzheimer's dementia. PIU was identified in 22.9% of FTD patients compared to only 0.8% of AD patients (*p* < 0.001). Behaviors included compulsive social media use, gaming, and online shopping. These findings suggest that PIU may represent an emerging behavioral feature associated with FTD, significantly more prevalent than in MCI and Alzheimer's dementia. Recognizing these digital behaviors could provide valuable clinical insights for diagnosis and management in the digital age.

## 1 Introduction

Frontotemporal dementia (FTD) is a neurodegenerative disorder within the spectrum of frontotemporal lobar degeneration (FTLD), primarily affecting the frontal and temporal lobes [1]. It encompasses the behavioral variant (bvFTD) and primary progressive aphasia (PPA), including the non‐fluent (nfvPPA) and semantic (svPPA) variants [2, 3]. Among these, bvFTD is characterized by early changes in behavior, personality, and social cognition, often accompanied by stereotyped, compulsive, or ritualistic behaviors [3]. Moreover, individuals with bvFTD show an increased vulnerability to addiction‐related behaviors [4], such as excessive alcohol and tobacco use [5].

The widespread accessibility of smartphones and the internet has led to an unprecedented rise in problematic online behaviors related to digital technologies, affecting individuals across all age groups [6]. Problematic online activities such as social media use have become a growing concern, with such behavior impacting daily functioning and interpersonal relationships [7]. However, it remains unclear whether patients with bvFTD, whose diagnostic criteria were developed before the ubiquity of Wi‐Fi‐enabled mobile devices (e.g., smartphones, tablets), are prone to developing these types of behaviors.

Given the intersection of bvFTD features, their potential susceptibility to addiction [5], and changes in social dynamics and complexity, understanding online behavior in this population is crucial. This study explores a series of bvFTD and PPA patients exhibiting problematic behaviors related to internet and social media use. By examining these cases, the aim was to provide clinical insight concerning the nature of such problematic behaviors among individuals with FTD and their clinical manifestations.

## 2 Methods

A retrospective review of patients diagnosed with MCI and Alzheimer's dementia (AD group), behavioral variant frontotemporal dementia (bvFTD), and primary progressive aphasia (PPA; nonfluent/agrammatic and semantic variants) was conducted at the Center for Neurodegenerative Diseases and the Aging Brain, University of Bari “Aldo Moro”, and the Pia Fondazione “Card. Panico” Hospital (Tricase). Diagnosis of AD was established according to the National Institute on Aging‐Alzheimer's Association (NIA‐AA) 2018 criteria [8]. bvFTD diagnosis was based on the consensus criteria proposed by Rascovsky et al. (2011) [3], and PPA diagnoses followed the classification by Gorno‐Tempini et al. (2011) [2], including the nonfluent/agrammatic (nfvPPA) and semantic (svPPA) variants. Patients were consecutively screened and enrolled in the study between January 1, 2023, and December 31, 2024. Demographic and clinical data were retrieved, including age, disease duration, education, Mini‐Mental State Examination (MMSE) scores, Clinical Dementia Rating (CDR) scores, and the presence of genetic FTD‐related mutations or repeat expansions [9].

In both cohorts, patients with a documented history of behaviors suggestive of problematic internet use (e.g., overuse, compulsivity, or dependency), were identified through clinical records and caregiver interviews. These patients underwent a semi‐structured interview specifically developed to assess patterns of internet, smartphone, and social media use, along with related behavioral changes. The interview, based on the biopsychosocial model of addiction proposed by *Griffiths* [10], explored the six core components of behavioral addiction: salience, mood modification, tolerance, withdrawal symptoms, conflict, and relapse. “Overuse” was operationally defined as a quantitatively excessive time spent online (often exceeding 12 h/day) disrupting daily routines; “Compulsivity” was defined as repetitive, ritualistic, or stereotyped digital behaviors lacking a functional goal (e.g., constant, non‐functional scrolling or repetitive video streaming); and “Dependency” was defined according to the aforementioned six‐component model. Additional domains included types of online activity, premorbid functioning, and perceived impact on personal and social life. Interviews were conducted separately with patients and caregivers, due to the frequent lack of insight observed in FTD patients. Full details of the interview protocol are provided in the Supporting Informations. All participants provided written informed consent prior to enrollment. The study was approved by the local ethics committee and conducted in accordance with the Declaration of Helsinki. Group comparisons used descriptive statistics and nonparametric tests (*χ* ^2^, Fisher's exact, and Mann–Whitney *U*; *p* < 0.05).

## 3 Results

Of the 61 patients evaluated in the FTD group, 14 (22.9%) exhibited problematic internet or social media use and were included in the study (Table 1). Among them, 10 (71.4%) had bvFTD, and 4 (28.6%) had PPA, including non‐fluent and semantic variants. Three carried pathogenic variants (two *C9orf72*, one *GRN*).

**TABLE 1:** Clinical and behavioral characteristics of patients with problematic internet use in Frontotemporal Dementia.

Case	Sex	Education, years	Diagnosis	Genetic variant	Disease duration, years	MMSE	Main features
1	Female	13	nfvPPA, sporadic	—	3	21	Binge‐watching; social isolation
2	Male	18	svPPA, sporadic	—	6	18	Problematic gaming; social isolation
3	Female	8	bvFTD, sporadic	—	3	22	Binge‐watching; problematic behavior on social media; social isolation
4	Male	16	nfvPPA, sporadic	—	4	21	Problematic social network usage; social isolation
5	Female	17	bvFTD, genetic	*GRN*	6	21	Problematic dating apps usage; problematic social network use
6	Male	13	bvFTD, genetic	*C9ORF72*	—	25	Problematic gaming; social isolation
7	Male	13	svPPA, sporadic	—	2	25	Social isolation; problematic social media browsing
8	Male	13	bvFTD, sporadic		5	24	Scrolling; online job platforms (LinkedIn…); social isolation
9	Male	17	bvFTD, sporadic	—	6	26	Scrolling
10	Male	13	bvFTD, genetic	*C9ORF72*	5	27	Problematic social media use; social isolation
11	Male	8	bvFTD, sporadic		1	17	Problematic online shopping
12	Female	17	bvFTD, sporadic		2	20	Problematic gaming; social isolation
13	Female	17	bvFTD, sporadic	—	5	23	Problematic gaming
14	Male	5	bvFTD, sporadic		4	24	Problematic online shopping
15	Female	18	AD		5	28	Problematic online shopping
16	Male	5	AD		5	23	Internet related delusions
17	Male	17	AD		3	26	Problematic gaming

Abbreviations: AD, MCI and Alzheimer's dementia; bvFTD, Behavioral variant frontotemporal dementia; FTD, Frontotemporal dementia; MMSE, Mini‐Mental State Examination (global cognitive functioning); nfvPPA, Non‐fluent variant primary progressive aphasia; PPA, Primary progressive aphasia; svPPA, Semantic variant primary progressive aphasia.

In the AD control cohort (*n* = 354), three patients (0.8%) showed comparable behaviors. The frequency of these behaviors was significantly higher in the FTD group than in the AD group (*p* < 0.001). In the FTD group, patients with FTD and problematic internet and social media use were significantly younger (*p <* 0.001) and had an earlier disease onset compared to those without such behaviors (*p* < 0.001). No significant differences were found in education level, sex distribution, global cognitive functioning (MMSE), and disease severity (CDR) between the groups. Clinical subgroup analysis showed that bvFTD patients with PIU were significantly younger (*p* < 0.001) and had an earlier disease onset (*p* = 0.013), while PPA cases with PIU exhibited greater disease severity (CDR; *p* < 0.001). No other demographic or cognitive differences were observed.

Within the FTD problematic‐use group, excessive social media use was observed in 6 patients (42.9% of the problematic internet use group; 9.8% of the total FTD cohort), associated with sleep deprivation, religious obsessions, extensive dating app use that resulted in extramarital affairs and divorce, and excessive job portal browsing without actual job‐seeking, highlighting significant disruptions in daily life and interpersonal relationships. Problematic gaming activities were present in 4 cases (28.6% of the problematic internet use group; 6.6% of the total FTD cohort), with gaming sessions exceeding 12 h per day, associated with social withdrawal and severely disrupted daily routines. Binge‐watching was reported in 2 patients (14.3% of the problematic internet use group; 3.3% of the total FTD cohort), characterized by excessive, repetitive video streaming that interfered with daily activities. Online buying impulsivity was noted in 2 patients (14.3% of the problematic internet use group; 3.3% of the total FTD cohort), including engagement in online scams and unnecessary purchases. Overlapping problematic internet‐related behaviors were present in 7 patients (50% of the problematic internet use group; 11.5% of the total FTD cohort), with social isolation emerging as a pervasive consequence. Nine out of 14 patients reported preferring online interactions over face‐to‐face communication, further exacerbating their withdrawal from social life. Six out of 14 patients showed frustration and anger when caregivers tried to limit their smartphone usage. In the AD group, problematic internet behaviors included compulsive social media use and online shopping (*n* = 1), AI‐related delusions (*n* = 1), and smartphone gaming (*n* = 1), each interfering with daily life and social functioning. Detailed case descriptions are provided in the Supporting Information.

## 4 Discussion

Problematic internet use may be a common behavior in the early stages of FTD. This case series highlights how the increasing prevalence of digital technology may influence the expression of neuropsychiatric symptoms among FTD patients. As diagnostic criteria for FTD were developed before the widespread adoption of online technologies, it is plausible that new behavioral patterns, including online addictions, are emerging among this population. While meta‐analyses estimate a 6.0%–7.0% PIU prevalence among the general population [11, 12], the 0.8% frequency observed in the AD cohort is notably lower, contrasting with the 22.9% prevalence identified among those with FTD and reinforcing PIU as a specific behavioral feature of the latter. Subgroup analyses suggested divergent drivers across the FTD spectrum. Among those with bvFTD, PIU was associated with a younger early‐onset phenotype, potentially reflecting a generational influence or a specific behavioral trait independent of cognitive decline. In contrast, among those with PPA, PIU was associated with greater disease severity, suggesting that in language variants, these behaviors may emerge as a non‐verbal outlet to potentially compensate for the progressive decline in linguistic abilities and social communication.

Previous studies suggest that the same neural circuits implicated in substance addictions, involving reward, impulse control, and decision‐making, may also underlie problematic behaviors associated with internet use [5]. These circuits are known to be compromised in FTD, particularly in bvFTD, with behavioral alterations often characterized by impulsivity, disinhibition, and compulsivity [5]. The parallels between online addiction and more traditional forms of addiction support the hypothesis that these behaviors stem from shared neurobiological mechanisms [13, 14, 15].

The societal implications of these findings are significant because internet use has become increasingly pervasive [16]. Patients with FTD may be especially vulnerable to developing maladaptive online behaviors, complicating their clinical management, exacerbating social isolation, and increasing caregivers' burden. Indeed, caregiver interventions, such as restricting smartphone access, may reduce the severity of these behaviors but may also provoke agitation, as observed in some cases.

However, several limitations must be emphasized. Specific applications (e.g., *Facebook games*, the video game *Call of Duty*) were identified but not systematically recorded for all cases, limiting the analysis of individual platforms. The retrospective design and reliance on clinical records and caregiver report may lead to under‐, or over‐reporting of internet‐related behaviors. Longitudinal research is also warranted to explore how these behaviors evolve or diminish with disease progression. As society adapts to new technologies, it is crucial that clinical frameworks evolve to account for these changes, ensuring accurate diagnosis and effective management of patients with FTD in the digital age.

## Author Contributions

Daniele Urso, Giuseppe Volpe, Mark D. Griffiths, and Giancarlo Logroscino contributed to the conception and design of the study. Daniele Urso, Giuseppe Volpe, Agnese Valguarnera, Davide Vilella, Alessandra Vitulli, Valentina Gnoni, Alessia Giugno, and Eleonora Rollo contributed to the acquisition and analysis of data. Daniele Urso, Giuseppe Volpe, Mark D. Griffiths, and Giancarlo Logroscino contributed to drafting a significant portion of the manuscript or figures.

## Funding

This work was supported by Regione Puglia and CNR for Tecnopolo per la Medicina di Precisione: D.G.R. n. 2117 of 21.11.2018 (Grant CUPB84I18000540002).

## Conflicts of Interest

The authors declare no conflicts of interest.

## Supporting information


**Data S1:** acn370417‐sup‐0001‐Supplementary material.docx.

## Data Availability

The data that support the findings of this study are available from the corresponding authors upon reasonable request.
