# Supplementary material for: Problematic Internet Use in Frontotemporal Dementia: A Case Series
Source: Ann Clin Transl Neurol. 2026 May 19:10.1002/acn3.70417. Online ahead of print. doi: 10.1002/acn3.70417 (PMC13394868; doi:10.1002/acn3.70417)
Supplement: Supplementary file 1 — Data S1: acn370417‐sup‐0001‐Supplementary material.docx. [file ACN3-9999-0-s001.docx]

**Supplemental Online Content**

**Table of Contents**

**Semi-structured interview
Cases Description
Case 1
Case 2
Case 3
Case 4
Case 5
Case 6
Case 7
Case 8
Case 9
Case 10
Case 11
Case 12
Case 13
Case 14**

**Case 15**

**Case 16**

**Case 17**

This supplemental material has been provided by the authors to give readers additional information about their work.

**Semi-structured interview**

Given the lack of standardized scales assessing Internet misuse in an elderly population, and even more so the absence of an instrument specifically designed to evaluate Internet misuse in individuals with neurodegenerative diseases, it was necessary to develop a tailored semi-structured interview. This interview was designed based on the biopsychosocial model of addiction proposed by Griffiths (2005) and aimed to explore the six dimensions of behavioral addiction outlined in his framework.

Specifically, *salience* was assessed by inquiring about the number of hours spent online daily and whether Internet use occurred as the first activity in the morning, during meals, or before sleep. *Mood Modification* was explored by investigating whether smartphones were used as a form of self-medication, such as for relaxation. *Tolerance* was assessed by examining whether Internet and smartphone usage had increased over time. *Withdrawal symptoms* were explored by assessing emotional and/or physical effects when Internet use was suddenly reduced or discontinued. *Conflict* was investigated by asking both the participant and the caregiver whether concerns had arisen regarding online activities. *Relapse*s were also considered; however, in many cases, when Internet use ceased, it was due to the progression of the neurodegenerative disease affecting cognition, particularly executive functions, ultimately causing significant difficulties using devices such as smartphone or tablets independently.

Additionally, the semi-structured interview examined the type of online activities performed and premorbid functioning, investigating whether and how the individual had used the Internet and smartphones before the onset of the condition to determine whether any significant changes had occurred. Furthermore, the personal and social impact of Internet use was assessed, with particular attention to whether excessive engagement contributed to diminished in-person interactions and heightened social isolation.

The interview was conducted separately with both the patient and the caregiver, as patients often lack insight into their behaviors. This dual report approach also allowed for the identification of potential cognitive distortions regarding smartphone and Internet use.

**Cases Description**

**Case 1**

A 60-year-old woman with history of working as a homemaker was diagnosed with non-fluent Primary Progressive Aphasia (PPA). Her past medical history was unremarkable, and she was not accustomed to drinking or smoking. The first symptom reported by her husband was forgetting her brother’s phone number, followed by progressive executive dysfunction and apathy. Over time, her preference for food and sweets intensified, coinciding with the emergence of dysregulated smartphone use. She became fixated on browsing cooking recipes and binge-watching video recipes, initially spending 3-4 hours daily on her phone, which eventually escalated to 7-8 hours per day. Despite this extensive browsing, she rarely prepared the recipes she viewed. Her husband described her as being "enslaved" to her phone, prioritizing it over other forms of entertainment, such as watching television or engaging in conversations. Her behavior led to social isolation, prompting her caregiver to confiscate the phone. Interestingly, she did not protest its removal and instead redirected her attention to alternative household activities, demonstrating a degree of adaptability when deprived of her device. Neuropsychological assessment revealed a cognitive profile characterized by a multidomain amnestic deficit, with primary memory deficit. The patient also exhibited prominent behavioral and psychological changes, including marked apathy and anxiety.

**Case 2**

A 62-year-old male lawyer presented with progressive cognitive impairment and was diagnosed with Frontotemporal Dementia, specifically the semantic variant of Primary Progressive Aphasia (PPA-sv). Following his diagnosis, the patient exhibited increasing apathy, spending the majority of his time smoking cigarettes and playing games on his smartphone. His wife reported that he developed a strong preference for smartphone use over other leisure activities, such as watching television. His routine included reaching for his phone immediately upon waking and using it throughout breakfast. He initially spent around 5-6 hours per day on his phone, engaging primarily in casual games that were considered cognitively stimulating by his physician, but ultimately socially isolating himself. Over time, his wife intervened to reduce his screen time, limiting usage to 2-3 hours per day. Notably, the patient did not strongly resist these restrictions, as his smartphone use did not significantly interfere with his daily life. However, he did display a specific obsession with keeping his phone’s battery fully charged, becoming visibly anxious and irritable whenever the battery level dropped, indicating a fixation on maintaining control over this aspect of his device usage. Neuropsychological evaluation showed a cognitive profile characterized by a single domain amnestic deficit. He also presents marked apathy.

**Case 3**

A 61-year-old former merchant and team leader with a history of smoking and regular alcohol consumption exhibited progressive cognitive and behavioral decline has been diagnosed as bvFTD. The patient developed a pattern of dysregulated television viewing, frequently engaging in binge-watching sessions that extended from morning until bedtime. Initially, she used Facebook for approximately 2 hours daily, primarily to send greetings to various groups. However, inappropriate online interactions, including oversharing personal information and engaging in online arguments, led her family to deactivate her account. Initially, she reacted with frustration and indignation, but she gradually withdrew from social media. Her smartphone usage subsequently shifted to streaming TV shows, further reinforcing her preference for digital entertainment over social interaction. This transition led to increased social withdrawal, making it difficult for her family to engage her in outings or social events. Attempts to limit her behavior were met with anger and aggression. Additionally, she exhibited a marked preoccupation with maintaining a fully charged phone battery, becoming anxious whenever the battery level dropped. Her digital media usage significantly altered her daily routine, reinforcing isolation and disrupting her sleep patterns. Neuropsychological assessment revealed a cognitive profile characterized by a multidomain non amnestic deficit, with primary executive dysfunctions. The patient also exhibited apathy, aggressiveness, hyperorality, disinhibition and irritability.

**Case 4**

A 71-year-old retired social worker presented with progressive cognitive and behavioral decline. By 2023, his condition had worsened, particularly in terms of apathy, apraxia of speech, agrammatism, and dysprosody, leading to a diagnosis of Non-Fluent Primary Progressive Aphasia (nfvPPA) with apraxia of speech. The patient began using his smartphone increasingly in 2020, coinciding with the onset of language difficulties. Over time, his smartphone uses escalated, reaching approximately 10 hours per day, including during meals and at bedtime, leading to significant sleep deprivation. His primary activities involved WhatsApp, Facebook, and Instagram, as well as checking emails and browsing deal websites. Additionally, he engaged in online card games like Burraco but did not spend money on in-app purchases. According to his wife, the smartphone served as a substitute for verbal communication and a coping mechanism for his emotional distress. His increasing reliance on the device contributed to complete social withdrawal, as he actively avoided personal interactions and instructed friends not to contact him due to his communication difficulties. His affect was marked by severe apathy and a persistently depressed mood. Neuropsychological assessment revealed a cognitive profile characterized by a multidomain non amnestic deficit, with attentional and executive dysfunction and language impairment. The patient also exhibited prominent behavioral and psychological changes, including depression, irritability, aggressiveness and hyperorality.

**Case 5**

A 50-year-old woman diagnosed with behavioral Frontotemporal Dementia and presenting a mutation in progranulin gene (*GRN*) exhibited dysregulated digital behavior. Details regarding her clinical presentation and disease progression have been extensively documented in a case report by Michelutti et al. (2024). Here, we focus on her specific patterns of internet and social media use. At the age of 45, the patient demonstrated an increased preoccupation with her physical appearance, dyeing her hair platinum blonde and dramatically escalating her social media activity. She frequently posted self-portraits, curated personal photoshoots, and even produced and distributed calendars featuring herself in professional model-like poses. Her use of dating applications became uncontrolled, facilitating multiple extramarital affairs, ultimately leading to the dissolution of her marriage and loss of custody of her two children. This behavioral pattern persisted for at least five years without significant modification.

**Case 6**

A 48-year-old male metalworker with 13 years of education exhibited behavioral alterations, including disinhibition and lack of empathy. Neuropsychological evaluation revealed a single non-amnesic executive dysfunction, (impairment in language and executive dysfunctions) and genetic testing identified an expansion of the C9orf72 allele, leading to a diagnosis of bvFTD. The patient’s partner reported an increasing pattern of smartphone use over the past two years, with uncontrolled daily gaming sessions lasting at least 4-5 hours. The first signs of this behavior emerged when the partner, returning from work, found the patient preoccupied with gaming instead of attending to their 2-year-old daughter. Over time, the patient’s phone use escalated further, reaching up to 12 hours per day on his days off. He frequently played while walking, even stumbling in the street, and displayed aggressive reactions when the partner attempted to take the phone away. His excessive smartphone use contributed to increasing social withdrawal, as he prioritized gaming over social and couple activities.

At work, he was demoted due to decreased responsibilities but continued spending significant time on his phone. His partner described him as "anxious to use the phone," noting that upon returning home with a depleted battery, he would rush to charge the device and resume gaming immediately, without removing his jacket or bag. Although the partner acknowledged that he had always engaged in gaming, the uncontrolled nature of his behavior had become increasingly evident in recent years, further isolating him from social interactions.

**Case 7**

A 57-year-old male municipal employee with a diagnosis of sporadic semantic variant Primary Progressive Aphasia (svPPA) exhibited a new pattern of smartphone usage, reportedly exceeding 12 hours per day according to his caregiver. His primary activity involved Facebook, where he consistently checked and sent birthday greetings to his contacts. Additionally, he displayed an unusual interest in monitoring daily celebrity obituaries online. Over time, he became increasingly socially withdrawn and apathetic, losing interest in all activities except for smartphone use. Another notable behavior was his constant monitoring of home security cameras through a mobile app. His wife attributed his excessive phone use to the progression of his cognitive impairment, suggesting that he gravitated towards simple, repetitive digital tasks as a way to maintain a sense of competence and control. Neuropsychological assessment revealed a cognitive profile characterized by a multidomain amnestic deficit. The patient also exhibited hyperorality, apathy, aggressiveness and irritability.

**Case 8**

A 38-year-old patient diagnosed with bvFTD exhibited scrolling behavior on his smartphone. According to his caregiver, he spent up to 15 hours daily scrolling and checking job portals without taking any steps to secure new employment. Colleagues also reported that the patient frequently and obsessively checked his phone during work hours. Approximately 4–5 years ago, the patient developed a compulsive smoking habit, which coincided with the onset of an increasing preoccupation with work. This preoccupation manifested as persistent inquiries about others' occupations and an excessive focus on job-seeking behaviors through online platforms. Over time, the amount of time spent on his smartphone increased significantly and it has impacted on his personal life, as he now prioritizes its use over engaging in activities with his son or socializing with others. Neuropsychological assessment revealed a cognitive profile characterized by a multidomain amnestic deficit, with probable memory impairment secondary to attentional and executive dysfunction. The patient also exhibited prominent behavioral and psychological changes, including apathy, irritability and anxiety.

**Case 9**

The wife of a patient 69-year-old with bvFTD reports a noticeable increase in her husband's smartphone use. Neurological examination suggests that he is in a prodromal stage of the disease, exhibiting early behavioral symptoms, including subtle but evident changes in mental rigidity. Neuropsychological evaluation highlights a single domain amnestic dysfunction. Notably, he has developed a greater reliance on his smartphone, using it to fill idle moments. While his total screen time is approximately three and a half hours per day, this represents a clear shift compared to a few months ago. The wife also observes that he increasingly struggles to follow movies, instead engaging in continuous scrolling through social media. Moreover, he exhibits heightened anxiety and frustration when unable to access his phone, suggesting that this early disease stage may be accompanied by emerging problematic smartphone use.

**Case 10**

The spouse of a 69-year-old patient diagnosed with behavioral variant frontotemporal dementia (bvFTD) reports a progressive increase in his smartphone usage. He spends most of the day scrolling through social media, engaging in repetitive behaviors such as liking posts and participating in online prayer rituals. His social media homepage is dominated by religious imagery, particularly representations of saints accompanied by prayers, as he becomes increasingly obsessed with religion. He appears to derive reinforcement from the auditory feedback of linking and scrolling, persisting in this behavior for extended periods. Additionally, he has fallen victim to online scams, losing over 250 euros, and is highly susceptible to digital advertising, regularly spending 60–70 euros per month on dietary supplements promoted through social media banners despite lacking a medical need. His caregiver faced frustration and anger when attempting to limit his smartphone usage. His social interactions have also diminished, as he now prefers engaging with his smartphone over interacting with visitors. Interestingly, both the patient and his sister carry the *C9orf72* mutation and have been diagnosed with behavioral frontotemporal dementia. Neuropsychological evaluation showed cognitive inhibition deficit, with larger executive dysfunction and amnestic deficit. He also showed apathy, anxiety and aggressiveness. While the patient developed problematic social media use and online spending, his sister exhibited a distinct yet similarly novel digital behavior, characterized by a progressive increase in online shopping, something entirely absents in her premorbid period. However, due to the advanced stage of her disease, she is now institutionalized and no longer able to use a smartphone. The emergence of these distinct but related online behaviors in both siblings suggests a potential link between FTD-related neurodegeneration and alterations in internet and digital engagement.

**Case 11**

A 51-year-old patient diagnosed with behavioral variant frontotemporal dementia (bvFTD) began to engage in increasing online shopping behavior, a pattern that was absent in the premorbid period. Previously, she used the internet in a functional manner, but over time, her behavior shifted to dysregulated scrolling through Facebook groups dedicated to selling used items, as well as local online platforms for second-hand goods. This new pattern of behavior led to her accumulating items, some of which were free, broken, or of no value. The situation eventually required her family to clear out her apartment. This shift in online usage emerged alongside the initial behavioral changes associated with her bvFTD diagnosis. Neuropsychological testing revealed a multidomain amnestic dysfunction characterized by severe executive and visuospatial impairment. Since last year she develops behavioral disturbances characterized by disinhibition, severe apathy and emotional detachment.

**Case 12**

A 44-year-old patient diagnosed as bvFTD gradually exhibited behavioral changes, including apathy, hyperorality, aggressiveness, and obsessive symptoms, which coincided with an increased reliance on her smartphone. Her husband reported that she would spend the entire day on her phone, engaging with social media and gaming platforms. She often isolated herself, prioritizing her phone over in-person interactions. Attempts by her relatives to limit her smartphone use were met with aggressive reactions. Neuropsychological testing revealed a multidomain amnestic dysfunction characterized by executive impairment with probable secondary memory deficits, consistent with a major neurocognitive disorder. As her condition advanced, however, her interest in gaming and social media diminished, likely due to the progression of her cognitive decline. Genetic screening is currently underway to better characterize her condition.

**Case 13**

Gaming appears to be one of the most frequent behaviors observed in patients with bvFTD. In this case, although the 62 years old patient engaged in social media use, her predominant activity was gaming. Over time, she began to dedicate increasing amounts of time to this activity. While her husband was unable to estimate the total hours spent gaming, he recalled that she would often play for at least four hours after they went to bed, despite his repeated requests to turn off the phone, as he was disturbed by the screen’s brightness. As the disease progressed, her interest in gaming gradually declined, consistent with the worsening of her cognitive condition. The patient shows a multiple amnestic cognitive disfunction, with apathy, disinhibition and hyperorality.

**Case 14**

The wife of a 61-year-old former merchant diagnosed as bvFTD reported that her husband spent excessive amounts of time on his smartphone, primarily engaging in uncontrolled online shopping. He frequently made purchases online, often spending hundreds of euros, despite her efforts to limit his behavior. However, as he remained functionally intact and lacked insight into the issue, controlling his spending proved challenging, leaving his wife feeling desperate. In an attempt to manage the situation, his wife was in discussions with their bank to establish a daily spending limit on his account. However, she faced resistance from her husband, who reacted with frustration and anger whenever his ability to make purchases was questioned, despite having also been a victim of online scams. Neuropsychological profile reveals multidomain amnesic deficit, with secondary memory impairment. He also presents apathy, aggressiveness, irritability, disinhibition and hyperorality.

**Case 15**

The daughter of a woman with atypical Alzheimer’s disease (frontal presentation) reported that her mother spent approximately eight hours per day engaged in compulsive scrolling. Most of this time was dedicated to Facebook, where she was repeatedly exposed to advertisements and purchased weight-loss supplements without medical indication. According to family members, the financial consequences were substantial, ultimately requiring them to block her credit card. In addition, frequent binge-watching episodes were observed, during which the patient was unable to disengage from the screen or perform other activities without a device present. Attempts to restrict her smartphone and internet use consistently provoked agitation and aggression. Neuropsychological assessment confirmed a major neurocognitive disorder with a predominantly dysexecutive profile, including secondary memory impairment. Behavioral features included apathy, disinhibition, and hyperorality

**Case 16**

An 82-year-old widowed man with mild cognitive impairment due to Alzheimer’s disease developed intense preoccupations centered on AI-generated videos of young women hosted on YouTube. Initially convinced the women were real, he later elaborated a delusional belief that they were “electromagnetic waves” able to enter and exit his smartphone at will, sometimes appearing as figures in his home. He spent most of his waking hours watching such videos, including while driving, waiting at appointments, and during the night, keeping his phone on the bedside table and waking repeatedly to “interact” with his “favorite woman.” The delusional system was elaborate, with episodes such as preparing dinner for the woman or seeking technical support to “release” her when she appeared “trapped” in the phone. Family attempts to dispute these beliefs provoked verbal aggression and occasional intimidation. Additional behaviors included mild disinhibition (sexually inappropriate remarks) and apathy. Despite preserving basic autonomy, his digital preoccupations significantly impaired quality of life and safety. He upgraded his devices and data plan to maintain uninterrupted access, prioritized smartphone use over social interactions and demonstrated anosognosia for these changes. Family members reported that a longstanding female acquaintance had introduced him to these videos; over time, his engagement escalated and became central to his daily routine.

**Case 17**

A 72-year-old man with mild cognitive impairment due to Alzheimer’s disease, presenting with a single-domain amnestic profile, developed marked changes in digital behavior. Alongside emotional lability, anxiety, and apathy, his wife reported that he spent approximately seven hours per day playing a smartphone-based card game. She described this as an obsessive pattern and a clear departure from his premorbid habits. The patient himself insisted that he could not stop playing, as he had achieved a high ranking on the online leaderboard. This behavior was associated with marital conflicts and withdrawal from previously valued activities, such as reading and gardening, in which he had long taken pleasure. His engagement with smartphone gaming progressively displaced social and leisure pursuits, reflecting an emerging pattern of problematic internet use.
